# Knowledge, attitude, and practice of physicians toward the management of diabetic ketoacidosis

**DOI:** 10.3389/fcdhc.2026.1723632

**Published:** 2026-02-04

**Authors:** Mabrouk AL-Rasheedi, Baharudin Ibrahim, Khawaja Husnain Haider, Ahmed Amin Ali, Hadzliana Zainal

**Affiliations:** 1 School of Pharmaceutical Sciences, University Sains Malaysia, Kuala Lumpur, Malaysia; 2 Albukairyah General Hospital, Ministry of Health, Al-Bukairyah, Saudi Arabia; 3 Department of Clinical Pharmacy, Faculty of Pharmacy, University of Malaya, Penang, Malaysia; 4 Department of Basic Sciences, Sulaiman AlRajhi University, Al-Bukairiyah, Saudi Arabia; 5 Department of Clinical Pharmacy, Faculty of Pharmacy, Kafrelsheikh University, Kafrelsheikh, Egypt

**Keywords:** attitude, diabetic, ketoacidosis, knowledge, physicians, practice

## Abstract

**Background:**

Diabetic ketoacidosis (DKA) is a serious complication of uncontrolled diabetes that results from insufficient insulin, high blood sugar levels, dehydration, and disturbances in acid-base status in the blood. This condition can arise from infections or treatment errors, such as inadequate insulin dosing or missed doses, and affects both type 1 and type 2 diabetes.

**Aim:**

The study aims to identify gaps in physicians’ knowledge, attitudes, and practices (KAP) regarding the updated Diabetic Ketoacidosis protocol from the Ministry of Health (MoH) to enhance educational programs and improve patient outcomes.

**Patients and methods:**

This cross-sectional study, conducted between July 2023 and July 2024, involved 242 physicians treating DKA across ten tertiary hospitals in five administrative regions of Saudi Arabia. Interns and physicians with less than one year of experience were excluded. Data were collected using a validated KAP questionnaire.

**Results:**

Among the 242 physicians surveyed, 57.9% demonstrated poor knowledge, 38.0% had fair knowledge, and only 4.1% exhibited good knowledge (Mean ± SD: 4.79 ± 1.85). Knowledge levels were significantly higher among females (p=0.001), physicians over 40 years of age (p<0.001), those with more than 15 years of experience (p<0.001), and endocrinologists (Mean ± SD: 6.25 ± 1.26, p<0.001). A favorable attitude was observed in 68.6% of physicians (Mean ± SD: 8.03 ± 1.81), significantly influenced by marital status (p=0.002). Good practices were noted in 51.7% of the physicians (Mean ± SD: 3.05 ± 1.84), with significant predictors including age, gender, experience, and region (p<0.05). The overall model explained 34.5% of the variability in knowledge, 16% of the variability in attitude, and 30.2% of the variability in practice.

**Conclusion:**

This study highlights significant gaps in physicians’ knowledge, attitudes, and practices regarding DKA management in Saudi Arabia. Younger, less experienced doctors tend to have lower knowledge scores, while higher scores are observed among endocrinologists and intensive care unit physicians, underscoring the need for targeted educational interventions.

## Introduction

a metabolic disorder characterized by impaired glucose oxidation resulting from insufficient insulin (1).

This condition is classified into Type 1 DM (T1DM) and Type 2 DM (T2DM). Diabetic ketoacidosis (DKA) is classically considered a complication of Type 1 Diabetes Mellitus (T1DM) due to absolute insulin deficiency. However, evidence shows that patients with Type 2 Diabetes Mellitus (T2DM) can also develop DKA, particularly under conditions of severe stress, infection, or relative insulin deficiency. In these cases, increased counter-regulatory hormones (glucagon, cortisol, catecholamines) and elevated free fatty acids promote ketogenesis, leading to metabolic acidosis (2).

Clinically, this association is important because physicians should not exclude DKA in patients with T2DM presenting with acute illness, poor glycemic control, or interruption of insulin therapy. Recognition of this link ensures timely diagnosis and management, reducing morbidity and mortality (3).

According to the review of the 10th Edition of the International Diabetes Federation (IDF), Atlas, 537 million adults worldwide have diabetes, projected to reach a colossal 783 million by 2045 (4, 5). This alarming trend, particularly in low- and middle-income countries, is burdening their healthcare systems due to high morbidity and mortality rates (6). The Middle East and North Africa region (MENA) carried the highest prevalence of diabetes in 2019 at 12.2% and is expected to witness a 96% increase in diabetes prevalence by 2045 (7).

The urgency of this situation, akin to a looming storm, cannot be overstated, and healthcare professionals must be aware and prepared for the impending challenges. A cross-sectional study conducted between 2007 and 2009 in Saudi Arabia, involving 18034 individuals over the age of 30, reported a diabetes prevalence of 25.4%, with 10.2% of those cases being previously undiagnosed. This prevalence was significantly higher in urban areas than rural ones (8).

If left unchecked, the situation may worsen due to serious complications associated with uncontrolled DM, with DKA being one of the most common. DKA arises from insufficient insulin, elevated blood sugar levels, dehydration, and increased acidity. Infections or treatment errors, such as inadequate insulin dosing, missed doses, or issues with insulin delivery, are important risk factors (9, 10).

Additional contributing factors include digestive disorders, heart problems, inflammatory diseases, pancreatitis, and substance abuse. Abdominal pain, nausea, vomiting, and fruity odor on the breath are common symptoms among patients (11). Moreover, some patients may also experience the typical signs of DM, such as frequent urination and thirst (12). The mortality rate following a single episode of DKA is reported to be 5.2%, and it rises by 6-fold with five or more admissions of DKA (13).

Physicians should promptly initiate the appropriate management of DKA by conducting a thorough physical examination, obtaining essential metabolic parameters, and establishing a definitive diagnosis, followed by starting an appropriate suitable treatment plan to prevent complications (14, 15).

The aim of this study was to highlight the gaps in physicians’ knowledge, attitudes, and practices (KAP) concerning the recently released updated DKA protocol from the Ministry of Health (MoH). Assessing these factors is vital for enhancing educational programs for physicians and ultimately improving the management of DKA. The need for improved physician education is evident, and this study’s findings can catalyze continuous learning and professional development, ultimately leading to better patient outcomes.

## Methods

### Study design and participants

A cross-sectional study design was conducted during the period from July 2023 and to July 2024 among physicians treating diabetic patients having DKA in Saudi Arabia. Saudi Arabia. Due to the high prevalence of diabetes, all five administrative health regions (Eastern, Western, Southern, Northern, and Central) were included in the survey. Two tertiary hospitals were selected from each region (**Table 1**). The study was conducted at the 10 selected institutions representing the five regions of the kingdom. Any physician not dealing with diabetic patients, having less than one year’s experience, and who are currently in their internship year was excluded from the study (16, 17).

**Table 1:** Distribution of participating tertiary hospitals by region.

Region	First tertiary hospital	Second tertiary hospital
Central	King Salman Hospital	King Fahad Hospital
Northern	King Khaled Hospital	King Abdulaziz Hospital
Southern	King Fahad Hospital	Gazan General Hospital
Eastern	Dammam Medical City	King Khaled Hospital
Western	Ohud Hospital	King Faisal Medical City

The sample size was determined based on an estimated 93,966 physicians across different specialties. Of these, 25% worked in EDs and ICUs, giving a minimum sample size of 80 physicians at a 95% confidence interval, 0.05 alpha error, and 80% power. The sample was raised to 242 physicians.

### Data collection tool

The data collection tool was a designed questionnaire evaluated by five experts and validated through a pilot study. It consists of two sections: the first section contains sociodemographic characteristics (age, sex, marital status, level of education, years of experience, specialty, and seniority), while the second section comprises three subsections assessing the studied participants’ KAP. The knowledge subsection had ten questions, scoring 1 for correct answer and 0 for incorrect, with a total score of 10. Participants were classified according to their level of knowledge into good (8-10), fair (5-7) and poor (<5). The attitude subsection comprised five questions with a three-point Likert scale (disagree, neutral, and agree). Each question scored 0 for an unfavorable attitude, 1 for a neutral attitude, or 2 for a favorable attitude, with a total score of 10. Attitude was classified into favorable (8-10), neutral (5-7), and unfavorable (<5). The practice subsection consisted of three questions with a three-point Likert scale (disagree, neutral, and agree). Each question was scored 2 with a total score of 6. poor practice was scored as 0, neutral practice as 1, and good practice as 2. Participants were classified as having poor practice (scoring only 2 out of 6) or good practice if scoring three or more (18).

### Ethical considerations

The study was approved by the USM Ethical Committee and by the Ethics Committees of the respective hospitals in Saudi Arabia and followed the international ethical guidelines of the Declaration of Helsinki (19). All participants were informed of the purpose and nature of the study, the privacy and confidentiality of data, and that participation was voluntary. Informed consent was obtained from all participants before the study was initiated. This process involved explaining the study protocol, ensuring anonymity by representing all subjects with codes rather than their names, and allowing participants to freely ask questions about the study with the option to withdraw at will without any consequences.

### Statistical analysis

Data was coded and entered using the statistical package for the Social Sciences (SPSS) version 25. According to the distribution of variables using the Kolmogorov-Smirnov test, non-parametric quantitative data was summarized by median as measures of central tendency & range as measures of dispersion. In contrast, parametric quantitative variables were summarized using mean and standard deviation. Frequencies and percentages describe categorical variables. Independent sample t-test and Kruskal-Wallis test were used to detect significant differences between quantitative variables. Pearson correlation was used to assess the correlation between quantitative variables (20). Multiple linear regression analysis was performed to detect the independent contribution of different factors affecting the knowledge, attitude, and practice of Physicians; enter method was selected, and R2 was used to detect the amount of variance in knowledge, attitude, and practice accounted by predictors, included in the model. Significance is considered when p is below 0.05.

## Results

**Table 2** presents the sociodemographic characteristics of the physicians. Males accounted for 51.2% of the sample, while females represented 48.8%. The mean age was 35.5 ± 8.8 years, ranging from 21 to 61 years. Overall, 61.2% of the participants were Saudi physicians. As regards region (31.8% of physicians from the Central region, followed by (24.4%) from the Western region, and the lowest percentage from the Northern region (9.5%). Regarding educational degrees, Physicians had ‘Bachelor and Board’ constituted 28.1% and 26.4%, respectively; 25.2% of Physicians were GPs, 21.5% were Internists, 18.2% were ER Physicians,14.9% were family medicine Physicians, and the remaining 10.3% and 9.9% were from ICU and Endocrinology departments, respectively.

**Table 2:** Socio-demographic characteristics of the study sample.

Socio-demographic characteristics	Study sample (n =242)	
	No.	%
Sex		
• Male	124	51.2
• Female	118	48.8
Age		
• 20-30	87	36.0
• 30-40	95	39.3
• 40 or more	60	24.8
Marital status		
• Single	86	35.5
• Married	132	54.5
• Divorced	15	6.2
• Widow	9	3.7
Nationality		
• Saudi	148	61.2
• Non-Saudi	94	38.8
Region		
• Northern Region	23	9.5
• Southern Region	30	12.4
• Central Region	77	31.8
• Eastern Region	53	21.9
• Western Region	59	24.4
The highest level of education		
• Bachelor	68	28.1
• Diploma	46	19.0
• Master	25	10.3
• Doctorate	20	8.3
• Resident (Board)	64	26.4
• Fellowship	19	7.9
Specialty		
• GPs	61	25.2
• Emergency medicine	44	18.2
• Endocrinology	24	9.9
• Family Medicine	36	14.9
• Intensive care unit	25	10.3
• Internal Medicine	52	21.5
Seniority		
• General practice	41	16.9
• Resident	78	32.2
• Senior	39	16.1
• Specialist	41	16.9
• Consultant	43	17.8
Experience years		
• 1-5	99	40.9
• 5-10	66	27.3
• 10-15	27	11.2
• 15-20	27	11.2
• >20	23	9.5
Age (Years)		
Min. – Max.	21 – 61	
Mean ± SD	35.5± 8.8	

More than half of physicians (57.9%) had a poor level of knowledge, with a mean score of 4.79 ± 1.85, while 68.6% had a favorable attitude toward MOH guidelines in DKA treatment, and 51.7% had a good practice, with means scores of 8.03 ± 1.81 and 3.05 ± 1.84, respectively, **Table 3**.

**Table 3:** KAP scores among the studied healthcare professionals.

Parameter	Study sample (n =242)
Knowledge (Q1-Q10)	Percentage (%)
Poor	140 (57.9)
Fair	92 (38.0)
Good	10 (4.1)
Min. – Max.	0.0 – 8.0
Mean ± SD.	4.79 ± 1.85
Attitude (Q11-Q15)	
Unfavorable	21 (8.7)
Neutral	55 (22.7)
Favorable	166 (68.6)
Min. – Max.	2.0 – 10.0
Mean ± SD	8.03 ± 1.81
Practice (Q16-Q18)	
Poor	117 (48.3)
Good	125 (51.7)
Min. – Max.	0.0 – 6.0
Mean ± SD	3.05 ± 1.84

**Table 4** shows the factors affecting knowledge, attitude, and practice. Knowledge was significantly higher among females than males (5.19 ± 1.74), p=0.001; physicians aged more than 40 years (5.92 ± 1.29), p<0.001; ever-married physicians, p<0.001; physicians from eastern and western regions (5.55 ± 1.87 and 5.53 ± 1.38, respectively), p<0.001, and physicians with more than 15 years of experience (6.11 ± 1.28 for experience 16–20 and 5.74 ± 1.1 for more than 20 years experience), p<0.001. Non-Saudi physicians and those with a higher education (more than bachelor’s degrees) had a higher knowledge than other physicians (p<0.001). Similarly, endocrinologists and ICU physicians had higher knowledge (6.25 ± 1.26 and 6.12 ± 1.36, respectively), p<0.001, akin to the consultant and senior physicians (5.91 ± 1.52 and 5.1 ± 1.83, respectively), p<0.001.

**Table 4:** Factors affecting KAP among the healthcare professionals studied.

Parameters	Knowledge		Attitude		Practice	
	Mean ± SD	p	Mean ± SD	p	Mean ± SD	p
Gender						
Male	4.4 ± 1.88	0.001*	8.19 ± 1.87	0.169	3.39 ± 1.8	0.004*
Female	5.19 ± 1.74		7.86 ± 1.74		2.7 ± 1.82	
Age groups						
20-30	4.17 ± 1.83	<0.001*	7.80 ± 1.67	0.053	2.84 ± 1.96	0.272
31-40	4.63 ± 1.87		7.97 ± 1.98		3.06 ± 1.84	
41 or more	5.92 ± 1.29		8.45 ± 1.70		3.35 ± 1.62	
Marital status						
Single	4.07 ± 1.78	<0.001*	7.53 ± 1.84	0.002*	2.57 ± 1.89	0.02*
Married	5.05 ± 1.81		8.34 ± 1.75		3.33 ± 1.75	
Divorced	6.20 ± 1.21		8.60 ± 1.68		3.47 ± 1.85	
Widow	5.33 ± 1.73		7.22 ± 1.56		3.0 ± 2.0	
Region						
Northern region	4.57 ± 1.67	<0.001*	7.74 ± 2.34	0.775	3.22 ± 2.15	0.325
Southern region	4.23 ± 1.96		8.37 ± 1.71		3.57 ± 1.50	
Central Region	3.97 ± 1.77		7.92 ± 1.73		3.21 ± 1.73	
Eastern Region	5.55 ± 1.87		8.08 ± 1.91		2.68 ± 1.85	
Western Region	5.53 ± 1.38		8.07 ± 1.68		2.86 ± 1.95	
Years of experience						
0-5	4.09 ± 1.89	<0.001*	7.85 ± 1.69	0.232	2.83 ± 1.84	0.205
6-10	4.86 ± 1.73		7.91 ± 2.01		2.98 ± 1.97	
11-15	5.0 ± 1.92		8.19 ± 1.86		3.37 ± 1.74	
16-20	6.11 ± 1.28		8.56 ± 1.55		3.70 ± 1.73	
>20	5.74 ± 1.1		8.35 ± 1.97		3.09 ± 1.59	
Nationality						
Saudi	4.39 ± 1.87	0.001*	8.05 ± 1.72	0.844	3.22 ± 1.79	0.084
Non-Saudi	5.41 ± 1.64		8.0 ± 1.97		2.80 ± 1.90	
Level of education						
Bachelor	4.04 ± 1.78	<0.001*	7.74 ± 1.98	0.608	3.03 ± 1.85	0.406
Diploma	5.41 ± 1.57		7.85 ± 1.87		2.61 ± 2.20	
Master	4.52 ± 1.87		8.32 ± 1.65		3.12 ± 1.79	
Doctorate	5.0 ± 2.18		8.15 ± 1.76		3.45 ± 1.67	
Resident (Board)	4.88 ± 1.77		8.27 ± 1.64		3.05 ± 1.71	
Fellowship	5.74 ± 1.76		8.21 ± 1.9		3.74 ± 1.33	
Specialty						
GPs	4.18 ± 1.84	0.001*	8.07 ± 1.72	0.260	3.15 ± 1.88	0.063
Emergency medicine	4.64 ± 1.92		7.84 ± 1.78		2.68 ± 2.0	
Endocrinology	6.25 ± 1.26		8.33 ± 2.12		3.46 ± 1.82	
Family medicine	4.14 ± 1.73		7.92 ± 1.78		2.94 ± 1.66	
Intensive care unit	6.12 ± 1.36		8.72 ± 1.37		3.88 ± 1.39	
Internal medicine	4.75 ± 1.69		7.75 ± 1.98		2.75 ± 1.88	
Seniority:						
General practice	4.24 ± 1.87	p<0.001*	7.85 ± 1.73	0.008*	3.02 ± 1.94	0.001*
Resident	4.47 ± 1.92		8.04 ± 1.90		2.62 ± 1.94	
Senior	5.10 ± 1.83		7.79 ± 2.17		3.54 ± 1.73	
Specialist	4.44 ± 1.55		7.59 ± 1.50		2.54 ± 1.87	
Consultant	5.91 ± 1.52		8.81 ± 1.47		3.93 ± 1.10	

Values are presented as Mean ± Standard Deviation (SD). Values marked with an asterisk () indicate statistical significance at p < 0.05*. GPs, General Practitioners. Reference categories for categorical variables were used in statistical testing.

For attitude, only marital status and seniority had a significant effect on physicians’ attitudes toward MOH guidelines.

**Table 5**, Knowledge shows a moderate positive correlation with age and years of experience, and weak but significant correlations with attitude and practice.

**Table 5:** Correlation of KAP scores with different parameters.

Parameter	Age		Years of experience		Knowledge		Attitude		Practice	
	r	p	r	p	r	p	r	p	r	p
Knowledge	0.341	<0.001*	0.346	<0.001*			0.146	0.023*	0.137	0.033*
Attitude	0.118	0.067	0.128	0.047*	0.146	0.023*			0.322	<0.001*
Practice	0.099	0.124	0.139	0.031*	0.137	0.033*	0.322	<0.001*		

r = Pearson’s correlation coefficient; p = significance level. Values marked with an asterisk () indicate statistical significance at p < 0.05*.

- Attitude has a weak correlation with experience, and a stronger significant correlation with practice.
- Practice shows a weak correlation with experience, and the strongest correlation with attitude.

**Figure 1:**
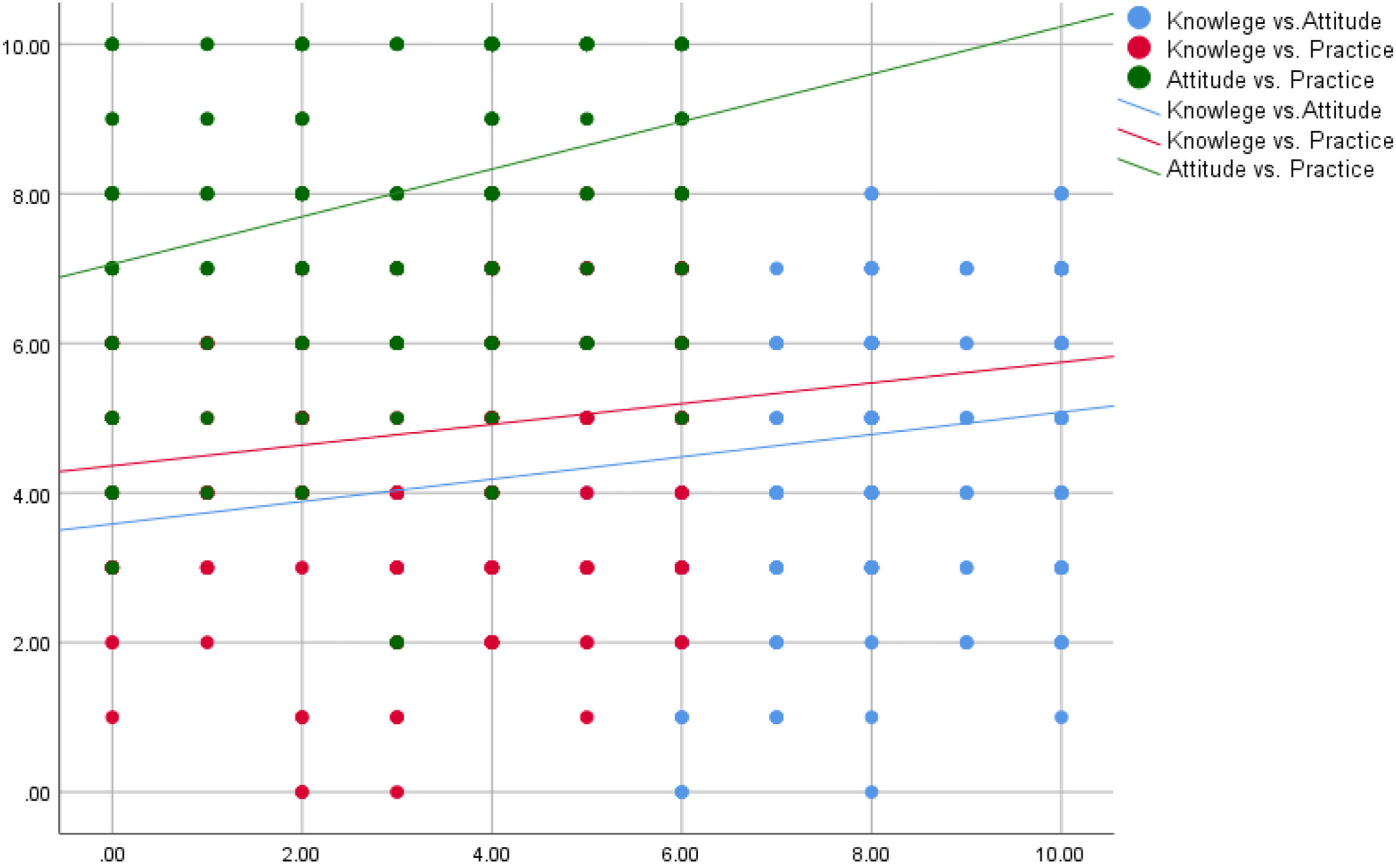
Correlation between KAP among physicians.

Knowledge correlated moderately with age and experience, while attitude showed the strongest correlation with practice (**Table 5**, **Figure 1**).

**Tables 6**, **7**, **8** illustrate three models to predict the effect of the independent contribution of different factors on KAP (**Table 6**). An overall model including sex, age, marital status, nationality, region, education, specialty, and seniority is statistically significant in predicting knowledge (F = 4.55, p<.001*). This Model explains 34.5% of the variability in the level of expertise of Physicians. Eastern region, Western region, and Endocrinology specialty are significant for contributing to the prediction of knowledge as b, t, p of these predictors, respectively [(.763, 2.338,.02) & (.660, 2.02,.045) and 1.15, 2.123, 035)]. At the same time, other factors did not significantly contribute to predicting knowledge.

**Table 6:** Multivariate analysis to predict the effect of the independent contribution of different factors on knowledge.

Predictors	Unstandardized coefficients		Standardized coefficients	t	Individual predictors sig	95.0% Confidence interval for B	
	B	Std. error	Beta			Lower bound	Upper bound
(Constant)	2.860	.825		3.467	.001*	1.234	4.486
Age (Years)	.023	.030	.108	.743	.458	-.037	.083
Gender “Female” ^a^	.168	.244	.045	.687	.493	-.313	.648
Years of experience	.014	.038	.054	.380	.705	-.060	.088
Nationality “non-Saudi”^b^	.303	.281	.080	1.079	.282	-.251	.858
Region ^c^							
Eastern Region	.763	.326	.171	2.338	.02*	.120	1.407
Northern Region	.189	.410	.030	.460	.646	-.620	.998
Western Region	.660	.327	.153	2.020	.045*	.016	1.304
Southern Region	-.217	.375	-.039	-.581	.562	-.956	.521
Marital status ^d^							
Married	.183	.295	.049	.620	.536	-.399	.764
Divorced	.692	.511	.090	1.354	.177	-.315	1.700
Widow	-.015	.616	-.002	-.024	.981	-1.229	1.200
Specialty ^e^							
Emergency medicine	.111	.461	.023	.241	.810	-.798	1.020
Endocrinology	1.150	.542	.186	2.123	.035*	.082	2.218
Family Medicine	-.136	.486	-.026	-.280	.780	-1.094	.822
Intensive care unit	1.037	.553	.171	1.875	.062	-.053	2.128
Internal Medicine	.073	.446	.016	.163	.870	-.807	.953
Seniority ^e^							
Resident	-.177	.462	-.045	-.383	.702	-1.088	.734
Senior	.385	.565	.077	.682	.496	-.728	1.499
Specialist	-.133	.541	-.027	-.245	.807	-1.200	.934
Consultant	.660	.682	.136	.967	.335	-.685	2.005
Level of education ^f^							
Diploma	.587	.394	.125	1.491	.137	-.189	1.364
Master	-.442	.457	-.073	-.967	.335	-1.342	.458
Doctorate	-.415	.546	-.062	-.760	.448	-1.490	.660
Resident (Board)	-.018	.364	-.004	-.049	.961	-.736	.700
Fellowship	-.426	.580	-.062	-.735	.463	-1.569	.716

Dependet Variable: knowledge

B = Unstandardized regression coefficient; Std. Error = Standard error of B; Beta = Standardized regression coefficient; t = t‑statistic; Sig = p‑value for individual predictors; CI = 95% Confidence Interval for B. Values marked with an asterisk () indicate statistical significance at p < 0.05*. a Reference category: female; b Reference category: Saudi nationality; c Reference category: Central Region; d Reference category: Single; e Reference category: General practice; f Reference category: Bachelor degree.

**Table 7:** Multivariate analysis to predict the effect of the independent contribution of different factors on attitude.

Predictors	Unstandardized coefficients		Standardized coefficients	t	Individual predictors sig	95.0% Confidence interval for B	
	B	Std. error	Beta			Lower bound	Upper bound
(Constant)	8.195	.942		8.697	.001*	6.338	10.053
Age (Years)	-.035	.034	-.168	-1.020	.309	-.101	.032
Gender “Female” ^a^	-.249	.271	-.069	-.918	.360	-.784	.286
Years of experience	.024	.042	.091	.565	.573	-.059	.106
Nationality “non-Saudi”^b^	-.163	.314	-.044	-.520	.604	-.781	.455
Region ^c^							
• Eastern Region	-.293	.368	-.067	-.797	.426	-1.018	.431
• Northern Region	-.621	.457	-.101	-1.361	.175	-1.521	.278
• Western Region	-.296	.367	-.070	-.808	.420	-1.019	.427
• Southern Region	.218	.417	.040	.523	.602	-.604	1.039
Marital status ^d^							
• Married	.825	.328	.227	2.513	.013*	.178	1.472
• Divorced	.532	.571	.071	.931	.353	-.594	1.657
• Widow	.121	.685	.013	.177	.860	-1.229	1.472
Specialty ^e^							
• Emergency medicine	-.829	.513	-.177	-1.617	.107	-1.840	.181
• Endocrinology	-.570	.609	-.094	-.936	.350	-1.769	.630
• Family Medicine	-.814	.540	-.160	-1.507	.133	-1.879	.251
• Intensive care unit	-.161	.620	-.027	-.259	.796	-1.383	1.062
• Internal Medicine	-.796	.496	-.181	-1.604	.110	-1.774	.182
Seniority ^f^							
• Resident	.536	.514	.138	1.041	.299	-.478	1.549
• Senior	.069	.629	.014	.109	.913	-1.171	1.308
• Specialist	-.144	.602	-.030	-.239	.811	-1.330	1.043
• Consultant	1.278	.760	.270	1.681	.094	-.220	2.777
Level of education ^g^							
• Diploma	.426	.440	.092	.967	.334	-.442	.426
• Master	.924	.509	.155	1.815	.071	-.079	.924
• Doctorate	-.212	.608	-.032	-.349	.728	-1.409	-.212
• Resident (Board)	.696	.405	.170	1.717	.087	-.103	.696
• Fellowship	-.267	.645	-.040	-.414	.679	-1.539	-.267
Knowledge	.107	.076	.110	1.420	.157	-.042	.107

B = Unstandardized regression coefficient; Std. error = Standard error of B; Beta = Standardized regression coefficient; t = t statistic; sig = significance level (p value); 95% CI = 95% confidence interval for B. Values marked with an asterisk () indicate statistical significance at p < 0.05*. Reference categories: a =gender, b = Saudi nationality, c = Central region, d = Single, e = General practitioners, f = General practice, g = level of education.

**Table 8:** Multivariate analysis to predict the effect of the independent contribution of different factors on practice.

Predictors	Unstandardized coefficients		Standardized coefficients	t	Individual predictors sig	95.0% Confidence interval for B	
	B	Std. error	Beta			Lower bound	Upper bound
(Constant)	3.152	1.014		3.107	.002*	1.152	5.151
**Age (Years)**	-.074	.031	-.357	-2.361	.019*	-.136	-.012
**Gender “Female” ^a^**	-.527	.252	-.144	-2.092	.038*	-1.023	-.030
**Years of experience**	.064	.039	.242	1.651	.100	-.012	.140
**Nationality “non-Saudi”^b^**	-.305	.291	-.081	-1.049	.295	-.878	.268
Region ^c^							
• Eastern Region	-.863	.341	-.194	-2.531	.012*	-1.534	-.191
• Northern Region	-.157	.425	-.025	-.371	.711	-.994	.679
• Western Region	-.649	.340	-.152	-1.909	.058	-1.320	.021
• Southern Region	.049	.386	.009	.126	.900	-.712	.810
Marital status ^d^							
• Married	.658	.308	.178	2.132	.034*	.050	1.265
• Divorced	.284	.530	.037	.537	.592	-.760	1.328
• Widow	1.253	.635	.129	1.975	.05*	.002	2.504
Specialty ^e^							
• Emergency medicine	-.880	.478	-.185	-1.843	.067	-1.822	.061
• Endocrinology	.180	.565	.029	.318	.750	-.933	1.293
• Family Medicine	-.199	.503	-.039	-.396	.693	-1.191	.793
• Intensive care unit	.154	.574	.026	.268	.789	-.978	1.286
• Internal Medicine	-.563	.462	-.126	-1.217	.225	-1.474	.348
Seniority ^f^							
• Resident	-.090	.477	-.023	-.188	.851	-1.031	.852
• Senior	1.195	.582	.239	2.052	.041*	.047	2.342
• Specialist	-.066	.557	-.013	-.118	.906	-1.164	1.033
• Consultant	1.312	.709	.273	1.851	.066	-.085	2.708
Level of education ^g^							
• Diploma	-.128	.408	-.027	-.314	.754	-.933	.677
• Master	-.381	.475	-.063	-.803	.423	-1.317	.554
• Doctorate	-.622	.563	-.093	-1.106	.270	-1.731	.487
• Resident (Board)	-.328	.378	-.079	-.868	.386	-1.073	.417
• Fellowship	-.420	.598	-.062	-.702	.483	-1.598	.759
**Knowledge**	.118	.070	.119	1.672	.096	-.021	.256
**Attitude**	.226	.063	.223	3.579	<.001*	.102	.350

Dependent Variable: practice. B = Unstandardized regression coefficient; Std. error = Standard error of B; Beta = Standardized regression coefficient; t = t statistic; sig = significance level (p value); 95% CI = 95% confidence interval for B. Values marked with an asterisk () indicate statistical significance at p < 0.05*. Reference categories: a = gender female, b =non Saudi nationality, c = Central region, d = Marital status, e = General practitioners, f = General practice, g = Level of education.

**Table 7** Over, all models, including sex, age, marital status, nationality, region, education, specialty, seniority, and knowledge level, are statistically significant in predicting attitude e as (F = 1.57, p=.044*), Model explains (16%) of variability in attitude score of Physicians. Married physicians are significant in predicting attitude as b, t, p (.825, 2.513, and.013, respectively), while other factors were non-significant.

(**Table 8**) Over, all models, including sex, age, marital status, nationality, region, education, specialty, seniority, knowledge level, and attitude, are highly statistically significant in predicting the practice of Physicians as (F = 3.427, p<.001*), Model explain (30.2%) of variability in practice of Physicians. Age, gender, years of experience, Eastern region, Married Physicians, widow Physicians, senior Physicians, and attitude are significant for predicting physicians’ practice as a p-value of these predictors <.05, while other factors were non-significant.

## Discussion

This current study aims to evaluate the KAP of physicians in Saudi Arabia in terms of DKA management. Considering the results, the present study provided several crucial observations. Most tertiary hospitals in Saudi Arabia have established standardized protocols for DKA management, and adherence to these protocols by physicians in emergency departments, intensive care units, and medical wards is essential to safeguard patient safety and uphold high standards of medical practice.

Most physicians had a low knowledge of DKA management, and more than half had a score below the cut-off mark. However, only 42% of physicians in this study understood the MOH guidelines on managing DKA, and only 68.6% of physicians had a good attitude toward the MOH guidelines on DKA management. On the same note, weak practice behavior was recorded in the management of DKA, with only 51.7% of the physicians recording good practice behavior.

We also explored the key factors that influenced the KAP scores. Specifically, our analysis revealed that knowledge scores were notably higher among female physicians, older physicians, and those with over 15 years of experience. Additionally, endocrinologists and ICU physicians demonstrated the highest levels of knowledge. In contrast, particularly those under 30 and with less than 15 years of experience had considerably lower knowledge levels. Interestingly, we found a strong correlation between knowledge and practice, as well as knowledge and attitude, suggesting that individuals with a more favorable attitude toward the process tend to possess adequate knowledge.

The results of Alemam (21) study agree with our research, indicating that lack of knowledge, inappropriate attitudes, and practices were reported in 57.2, 58.2, and 62.6% of the studied PCPs, respectively. The previous study mentioned that age below 32, being unmarried, having Bachelor’s or Master’s degrees only, working experience for less than 5 years, and being in a GP position were significantly correlated with lack of knowledge, which goes hand in hand with the present study.

Our study also shows that knowledge was significantly higher among physicians with more than 15 years of experience, an observation supported by Onyiriuka et al. (22) who reported that a significantly higher proportion (36.7%) of physicians who have practiced for more than 10 years answered questionnaires correctly compared with their counterparts (22.2%) whose medical practice was 10 years or less.

Hepprich et al. discovered that 32% of Type 1 diabetic patients were unaware of DKA, and nearly half failed to identify either a symptom or a cause of DKA. This lack of knowledge correlated with the inadequate understanding of DKA management exhibited by more than 57% of physicians in the current study. Despite our research focusing on different groups—patients in their study and healthcare providers in ours—there exists an issue with DKA education throughout the continuum of care. Interestingly, Hepprich et al. also noted an overestimation of patients’ understanding of DKA and highlighted similar information deficits among healthcare professionals. These data imply variations in the knowledge and updates of healthcare providers. Also, the patients themselves may not have the technical expertise to manage DKA appropriately, indicating a need for continuous training. Our study is consistent with this line of research. It emphasizes the need for continuous, multi-faceted educational approaches to address the knowledge deficit and enhance DKA management results in alignment with the study of Hepprich et al. (23).

Hassan et al. reveal significant deficiencies in understanding DKA among diabetic patients and healthcare professionals. Only 42% of participants recognized DKA as an emergency, and merely 33% identified polydipsia as a key symptom. This concurs with our data that over half of the physicians had inadequate knowledge about DKA and its treatment, highlighting a widespread lack of awareness in the patient and medical communities. It is pertinent to mention that Hassan et al. primarily focused on diabetic patients from the northern and western regions of Saudi Arabia. Nevertheless, similar to Hassan et al. we emphasized the need for targeted educational initiatives to enhance clinicians’ understanding of DKA. Notably, Hassan et al. also investigated knowledge of DKA prevention and management among patients presenting in emergency departments. They also observed that many patients allowed to manage DKA believed that frequent self-monitoring of blood sugar was the best preventive strategy. This underscores the need for targeted, multi-faceted educational approaches to address these knowledge deficits and enhance DKA management results in alignment with the published data (24).

A cross-sectional study in the central region of Saudi Arabia aimed to assess the knowledge, attitudes, and practices related to diabetes among patients diagnosed with the condition. The findings indicated that the patients exhibited generally high knowledge scores (73.6%) and positive attitude scores (87.7%). However, their practice scores were notably low at 45%. The authors identified a moderate positive correlation between self-knowledge and attitude (r = 0.503, P < 0.001), as well as between knowledge and practice (r = 0.337, P < 0.001). Additionally, there was a lower positive correlation between attitude and practice (r = 0.235, P < 0.001). However, their poor practice scores show a pressing need to design better diabetes education programs that improve their knowledge and lead to improved practices in managing diabetics. This disparity between understanding and performance underlines the urgency for comprehensive patient education emphasizing self-management, to manage diabetic complications (25).

### Knowledge gaps among physicians

According to our data, Elghamrawy et al. reported that although the majority recognize the typical symptoms of DKA, and select the appropriate therapeutic options for effectively managing DKA, there were still some gaps in knowledge, as a sizeable portion of the participants were unable to accurately identify several of the essential characteristics of DKA (26). Also, Madkhly et al. found that the majority of medical students had basic knowledge about diabetes, including its clinical aspects and therapy. On the other hand, there were certain knowledge gaps about DKA, however 76% were aware of the mode of insulin administration (27).

Similarly, Singh et al. assessed medical officers in primary health facilities. They found that while they had a solid understanding of T2DM, their knowledge of DKA was lacking, with only 50% recognizing its general characteristics and treatment. Despite the information deficiencies, our survey found that 68.6% of physicians had a positive disposition toward MOH recommendations for DKA care (28). This commitment to following established norms and guidelines for enhancing patient outcomes is reassuring and underscores the dedication of healthcare providers.

Powers et al. recognized diabetes as a significant public health issue, they emphasize that diabetes self-management education and support is crucial for effective diabetes management, yet there is a low participation rate in these programs (29). Specifically, many providers have misunderstandings about the necessity and effectiveness of self-management education and support, and they may have difficulty identifying when and how to make referrals. they cited that only 6.8% of privately insured individuals with newly diagnosed type 2 diabetes participated in diabetes self-management education and support within 12 months of diagnosis, and only 5% of Medicare participants receive diabetes self-management education and support during their first year of diagnosis. This low acknowledgment of diabetes self-management education and support as an essential component of diabetes treatment underscores a significant gap in care, highlighting a disparity between perception and a thorough comprehension of integrated care techniques (30). Our data reveals that merely 51.7% of physicians exhibited proficient practice in managing DKA despite a predominantly favorable disposition toward MOH standards. This divergence between attitude and practice underscores possible obstacles in applying information to clinical practice.

Hamelin et al. reported that majority of physicians reported the guidelines to be useful (83.6%); 54.6% of respondents were familiar with the guidelines, and 54.7% claimed to use them in clinical practice, thus underscoring the significance of resource access in knowledge application to practice (31). Although physicians possess a good comprehension of T2DM-related theory, the practical application of this information, especially about diagnostic criteria, such as HbA1c, is inadequate due to insufficient facilities and resources at these centers (32).

This corresponds with our observation that although physicians acknowledge the significance of specific tests and procedures, logistical limitations frequently hinder their ability to implement these procedures. Furthermore, physicians possessing higher knowledge ratings were more inclined to demonstrate positive attitudes and superior clinical practices (33). Our study revealed a favorable correlation among knowledge, attitude, and practice ratings, indicating that an increase in knowledge may enhance attitudes and, in turn, improve clinical practices in DKA care.

### Implications for clinical practice and policy

The findings of this study have clinical significance for managing DKA in Saudi Arabia. Identified gaps highlight an urgent need to enhance KAP related to this life-threatening condition. More than half of the physicians demonstrate a knowledge gap, underscoring the urgency for continuous education and development initiatives. These efforts should target various medical specialties, particularly first-line physicians who are most likely to encounter these cases, and subsequently refer them to specialists, such as endocrinologists. Training should extend beyond general principles of DKA management to include practical decision-making skills, ensuring that physicians are fully equipped to handle DKA in real-world scenarios.

## Recommendations and future directions

At the policy level, healthcare unit administrators should consider implementing uniform policies to manage DKA across various facilities. This approach would ensure that all patients receive the same high-quality care, regardless of location. Furthermore, incorporating regular audit and feedback cycles within the healthcare organization could help assess adherence to guidelines for managing DKA patients.

Based on the data from this study, physicians in Saudi Arabia have proposed several recommendations for future research and enhancements in managing DKA. There is a noticeable absence of comprehensive educational programs, which should be an essential component of continuing medical education. These programs should focus on theoretical knowledge and practical skills, particularly for emergency medicine physicians. To prevent inconsistencies or inefficiencies in DKA management, the Ministry of Health (MOH) should establish standardized clinical management guidelines for DKA, which should be regularly updated and disseminated to all clinics nationwide.

Additionally, targeted training initiatives should be implemented for high-risk physicians, especially those who are young and inexperienced, through mentorship programs involving qualified consultants in endocrinology and intensive care. Regular evaluations of DKA management practices within healthcare organizations should be conducted to assess compliance with clinical guidelines, using the results to enhance training efforts. Furthermore, it is crucial to develop patient education programs that improve understanding of DKA prevention and early identification, complementing the educational efforts of physicians.

## Limitations

The primary limitation of this study is its reliance on cross-sectional study data, which captures information at a single point in time. As a result, it does not provide insights into changes in knowledge, attitudes, and practices (KAP) over time or reflect the evolving awareness within the community. Additionally, this research is based exclusively on the perceptions of the respondents, which raises the possibility of socially desirable responses; thus, participants may report a level of knowledge or adherence to guidelines that exceed their actual practices. Furthermore, the study’s participants were limited to medical specialists with at least two years of clinical practice and no general practice experience. This constraint in the study population might have hindered the generalization of the findings to the broader population of physicians.

## Conclusion

The findings of this study offer a thorough evaluation of physicians’ KAP regarding the management of DKA in Saudi Arabia. The results indicate a significant lack of understanding and poor adherence to the best practices surrounding DKA; over half of the physicians misinterpreted at least one aspect of the condition, while the remaining physicians demonstrated favorable management practices. Such shortcomings are of concern, particularly given the severity of DKA and the associated high risk of morbidity and mortality. Factors such as age, experience, and specialty served as predictors, revealing that endocrinologists and ICU physicians exhibited higher levels of knowledge. In contrast, younger, less experienced physicians showed knowledge gaps in several key areas.

## Data Availability

The original contributions presented in the study are included in the article/supplementary material. Further inquiries can be directed to the corresponding author.
